# Social-group identity and population substructure in admixed populations in New Mexico and Latin America

**DOI:** 10.1371/journal.pone.0185503

**Published:** 2017-10-04

**Authors:** Meghan E. Healy, Deirdre Hill, Marianne Berwick, Heather Edgar, Jessica Gross, Keith Hunley

**Affiliations:** 1 Department of Anthropology, University of New Mexico, Albuquerque, NM, United States of America; 2 Department of Internal Medicine, University of New Mexico, Albuquerque, NM, United States of America; Universitat Pompeu Fabra, SPAIN

## Abstract

We examined the relationship between continental-level genetic ancestry and racial and ethnic identity in an admixed population in New Mexico with the goal of increasing our understanding of how racial and ethnic identity influence genetic substructure in admixed populations. Our sample consists of 98 New Mexicans who self-identified as Hispanic or Latino (NM-HL) and who further categorized themselves by race and ethnic subgroup membership. The genetic data consist of 270 newly-published autosomal microsatellites from the NM-HL sample and previously published data from 57 globally distributed populations, including 13 admixed samples from Central and South America. For these data, we 1) summarized the major axes of genetic variation using principal component analyses, 2) performed tests of Hardy Weinberg equilibrium, 3) compared empirical genetic ancestry distributions to those predicted under a model of admixture that lacked substructure, 4) tested the hypotheses that individuals in each sample had 100%, 0%, and the sample-mean percentage of African, European, and Native American ancestry. We found that most NM-HL identify themselves and their parents as belonging to one of two groups, conforming to a region-specific narrative that distinguishes recent immigrants from Mexico from individuals whose families have resided in New Mexico for generations and who emphasize their Spanish heritage. The “Spanish” group had significantly lower Native American ancestry and higher European ancestry than the “Mexican” group. Positive F_IS_ values, PCA plots, and heterogeneous ancestry distributions suggest that most Central and South America admixed samples also contain substructure, and that this substructure may be related to variation in social identity. Genetic substructure appears to be common in admixed populations in the Americas and may confound attempts to identify disease-causing genes and to understand the social causes of variation in health outcomes and social inequality.

## Introduction

Admixed populations form when individuals from previously separated populations mate with one another. Research on admixed human populations can assist in detecting alleles underlying susceptibility to common diseases [1–3], and it can provide important insights into the causes of social inequality and health disparities in societies that are stratified by race or ethnicity [4,5]. Population substructure is potentially an important confound in this research. Substructure occurs when individuals within a larger population mate assortatively due to the presence of geographic barriers, or to structured racial, ethnic, linguistic, or phenotypic variation. Failure to recognize and account for substructure can lead to spurious conclusions about genetic and social causes of disease [4], and it can lead to suboptimal distributions of resources intended to ameliorate the pernicious consequences of racial and ethnic discrimination.

In this paper, we examine substructure associated with racial and ethnic identity in admixed populations in the Americas, concentrating on the “Hispanic or Latino” (HL) population of New Mexico. We chose to focus on New Mexico for three reasons. First, it has the highest proportion of any US state of individuals that identify as HL on the US Census (47% in 2010) [6]. Second, historical and demographic records provide detailed information about the formation of heterogeneity in social identity within the HL population of the state over the past 400 years [7,8]. Third, previous genetic studies have documented the existence of genetic substructure in HL populations in the region [9,10]. These factors make the HL population of New Mexico ideal for studying the relationship between population substructure and social-group identity in the US, and for drawing inferences about this relationship in other admixed populations in the Americas.

### Background: Social-group identity in New Mexico

When the first Spanish colonists arrived in the area that would become New Mexico in 1598, they encountered Native Americans whose ancestors had been present in the Americas for more than 13,000 years [11]. The earliest Spanish census and church records enumerated individuals of mixed ancestry as well as exclusive Spanish or Native American ancestry [12,13], indicating that mating between Spaniards and Native Americans occurred from earliest contact [12–15]. These records show that, during the colonial period (1598–1821), Spaniards used a hierarchical caste system to describe and socially rank admixed individuals according to the fraction of Spanish and Native American heritage. During the US territorial period (1850–1912), the population of New Mexico grew as people migrated from other areas of the US. In the face of potential political marginalization by the new arrivals, the resident population re-embraced its Spanish, i.e., European, roots. Later, in response to increasing migration from Mexico, many New Mexicans began to embrace and romanticize the Spanish heritage of the long-resident population [8,16]. The rate of migration from Mexico increased in 20th century, and today more than half of New Mexicans who identify themselves as HL on the US census also identify as Mexican or Mexican American [17]. As a result of this region-specific history, the notion of comparatively recent Mexican vs. deep Spanish heritage is a salient feature of social identity in New Mexico today [8].

Here, we use four complementary statistical methods to document the existence of genetic substructure in fourteen HL populations with widespread distribution throughout the Americas, and we use the HL population of New Mexico to examine the role played by racial and ethnic identity in formation of this substructure.

## Materials and methods

### Ethics statement

The University of New Mexico Institutional Review Board approved the protocols used to conduct this research (UNM IRB 98–369). Written informed consent was obtained from all participants prior to data collection.

### Terminology

In 1997, the Office of Management and Budget began to use the phrase “Hispanic or Latino” to refer to US residents of Spanish origin or descent, defined as “of Cuban, Mexican, Puerto Rican, South or Central American, or other Spanish culture or origin regardless of race” [18]. The two terms in the phrase were meant to capture regional variation in the US, and in principle, the term “Latino” includes individuals of Portuguese origin, e.g., from Brazil. Based on these conventions, in the current study, we use the term “NM-HL” to refer to the New Mexican HL sample. Additionally, while noting that the two terms are not widely used outside of the US [19], we use “LA-HL” to refer to people of mixed African, European, and Native American ancestry in Latin America.

### Self-identified race and ethnicity

Our primary NM-HL sample consists of 98 unrelated adult individuals recruited from the Cancer Genetics Network (CGN) [20], which consists of cancer patients and their first-degree relatives. During initial recruitment for the CGN, telephone interviewers used two questions to elicit information about the ethnic and racial identity of participants and their parents. The first question was “How would you describe your ethnic or racial background?” Participants were asked to choose all that applied from the list:

- White
- Hispanic, Spanish or Latino
- Black or African American
- Native American, Aleutian, Eskimo
- Asian or Pacific Islander
- Other

Because the question contains both OMB race and ethnic categories, we refer to this self-classification as “ethno-racial.” We use the acronym “HSL” to refer to the choice “Hispanic, Spanish or Latino.”

The second question was “Would you describe yourself as…?” Participants were asked to choose all that applied from the list:

- Mexican, Chicano, New Mexican
- Puerto Rican
- Cuban
- South American or Central American
- Other specified Spanish origin (including European)
- Spanish, Hispanic, or Latino, not otherwise specified

Since these terms refer to subcategories of HL [18], we refer to this self-classification as “ethnic subgroup.” We use the acronym “MCN” to refer to the choice “Mexican, Chicano, New Mexican.” We address the potential limitations of these terms for NM-HL in the Discussion.

### Genetic data

We mailed DNA mouthwash kits to 159 individuals located throughout the state who minimally responded “Hispanic, Spanish or Latino” to the ethno-racial question. DNA extracts from 109 respondents were genotyped for 270 autosomal microsatellite loci from Screening Set 16 from the Marshfield Clinic’s Mammalian Genotyping Service [21]. Eleven individuals failed to genotype, for a final sample of 98 individuals (32 males and 66 females; 83 cancer patients and 15 unrelated controls). We matched these loci to published data from 1,062 individuals in seven African, eight European, 29 Native American, and 13 admixed LA-HL samples from Central and South America [22–24].

The published LA-HL data (Table 1) were used to assess the extent of genetic substructure in HL populations across the Americas. The data were collected for population genetic analyses or disease association studies [24]; the studies did not provide information about the racial or ethnic identity of the individuals. Data for twelve of the samples were collected in single locations in Mexico (Mexico City), Guatemala (Oriente), Costa Rica (Central Valley, abbreviated CVCR), Colombia (four samples from Peque, Medellin, Cundinamarca, Pasto), Chile (two samples from Paposo and Quetalmahue), Argentina (three samples from Salta, Tucuman, and Catamarca). Data for the Brazil sample (Rio Grande do Sul, abbreviated RGS) were collected in two cities (Bagé and Alegrete).

**Table 1:** HL sample sizes and locations.

Sample	n	Location
Mexico City	19	Mexico
Oriente	20	Guatemala
CVCR	20	Costa Rica
Pasto	19	Colombia
Peque	20	Colombia
Medellin	20	Colombia
Cundinamarca	19	Colombia
RGS	20	Brazil
Quetalmahue	20	Chile
Paposo	20	Chile
Catamarca	14	Argentina
Salta	19	Argentina
Tucuman	19	Argentina
New Mexico	98	USA

### Statistical methods

We used four complementary approaches to examine substructure in the HL samples. First, to explore gross structure among the populations, we summarized the major axes of genetic variation for the HL samples and NM-HL ethno-racial and ethnic subgroups using PCA. The analyses were conducted using the ade4 package [25] in R [26].

The second approach is based on the fact that structured populations have a reduction of heterozygosity relative to that predicted under HWE [27]. To look for this reduction, we calculated F_IS_ for each sample as the average across loci of the quantity *1 - (Observed Heterozygosity/Expected Heterozygosity)*. Positive values indicate a reduction of heterozygosity relative to that predicted under HWE. The analyses were conducted using GenePop [28].

The third approach is based on research by Verdu and Rosenberg [29] that demonstrated that, under a model in which two parental source populations contributed to an admixed population one time in the past, and mating was subsequently random with respect to genetic ancestry, variation in ancestry decreased every generation until all individuals had identical ancestry proportions. To test this “one-time,” random-mating model for the HL samples, we first estimated African, European, and Native American ancestry proportions for all individuals using the model-based clustering approach implemented in STRUCTURE [30]. We ran STRUCTURE at values of *K* from 2 to 15 using the published African, European, and Native American samples as parental sources. For each value of K, we ran STRUCTURE 10 times using a burn-in phase of 25,000 steps and 15,000 MCMC repetitions. Preempting our results, the average African ancestry in the NM-HL samples was low at 1.3%, and the average across the LA-HL samples was only 2.3%. To simplify the analyses of the one-time model, we only considered admixture between Europeans and Native Americans.

We then generated model-based genetic ancestry distributions in which, in the first generation, Europeans mated with one another in proportion 0.542, and Europeans mated with Native Americans in proportion 0.458; these values produced the mean European and Native American ancestry proportions in the NM-HL sample. Each subsequent generation, individuals in the admixed population mated randomly with respect to genetic ancestry. We ran the model 15 times for admixture onset dates of between 1–15 generations before the present. The 15-generation value corresponds roughly to the date 1598 AD, when Spaniards first arrived in New Mexico. These analyses were based on the “single event admixture” model described by Verdu and Rosenberg, in which the variance in ancestry at generation *g* is given by s*(1 –*s*)/2^*g*^, where *s* is the proportionate contribution from the European parental source population at the time of initial admixture. The model-based ancestry distributions were generated using an original R script written by one of the authors (JMG).

Based on these results, for our fourth approach, we used the 90% credible regions estimated in STRUCTURE to test the hypotheses that individuals in each HL sample had 100%, 0%, and that sample’s mean percentage of African, European, and Native American ancestry. We also tested for mean differences in African, European, and Native American ancestry between the NM-HL ethnic subgroups using signed Wilcoxon rank-sum tests.

## Results

In Tables 2 and 3, we show various combinations of choices for the two identity questions. For the ethno-racial question, all participants chose “Hispanic, Spanish, Latino”, and 20 participants also chose “White” (Table 2). None of the participants chose “Black or African American,” “Native American, Aleutian, Eskimo,” or “Asian or Pacific Islander.”

**Table 2:** NMS ethno-racial and ethnic subgroup identity.

Grouping	Categories	Self	Mother	Father	All three
Ethno-racial group	HSL	98	90	90	83
	HSL & White	20	11	6	3
	HSL & no race	78	79	84	73
	Non-HSL & White	0	8	6	0
	Non-HSL & no race	0	0	2	0
Ethnic subgroup	MCN	58	48	56	45
	Spanish	34	34	30	24
	Both MCN and Spanish	4	6	9	2
	Neither MCN nor Spanish	2	10	3	2

**Table 3:** Ethnicity-race, participants and parents.

	Mother			
Self	HSL & White	HSL & no race	Non-HSL & White	Non-HSL & no race
HSL & White	8	4	8	0
HSL & no race	3	75	0	0
				
	Father			
Self	HSL & White	HSL & no race	Non-HSL & White	Non-HSL & no race
HSL & White	5	8	6	1
HSL & no race	1	76	0	1
				
	Father			
Mother	HSL & White	HSL & no race	Non-HSL & White	Non-HSL & no race
HSL & White	3	5	3	0
HSL & no race	2	73	3	1
Non-HSL & White	1	6	0	1
Non-HSL & no race	0	0	0	0

For the ethnic subgroup question, we created a single “Spanish” subgroup by combining individuals who responded “Other specified Spanish origin (including European)” or “Spanish, Hispanic, or Latino, not otherwise specified.” We note that 27 of the 34 individuals in this subgroup chose the first of these two options. Fifty-eight individuals chose MCN (“Mexican, Chicano, New Mexican”) exclusively. Four individuals self-identified as belonging to both the Spanish and MCN subgroup, and two did not identify with either. None of the other ethnic subgroups were chosen by participants. Most participants placed one or both parents either exclusively in the Spanish subgroup or exclusively in the MCN subgroup.

Table 3 compares ethno-racial identity between the participant and each parent, and between the two parents. Most participants placed both parents into the same ethno-racial group that they chose for themselves. A notable exception is that 14 participants identified one parent as non-HSL and White. Of these participants, six identified themselves as MCN, six identified as Spanish, one identified as both, and one identified as neither. No participants identified both parents as non-HSL and White.

For the ethnic subgroup question, the vast majority of participants (> 93%) placed both parents into the same ethnic subgroup that they chose for themselves (S1 Table).

Based on these results, we divided the NM-HL sample into five subgroups for the analyses of the genetic data. The first two subgroups contain participants who self-identified exclusively as either Spanish or MCN. The second two subgroups are subsets of the first two; they consist of individuals that identified themselves and both parents as belonging exclusively to one of the subgroups. We refer to these two groups as “All Spanish” and “All MCN.” The fifth subgroup consists of participants who identified one parent as non-HSL and White.

### PCA

**Fig 1:**
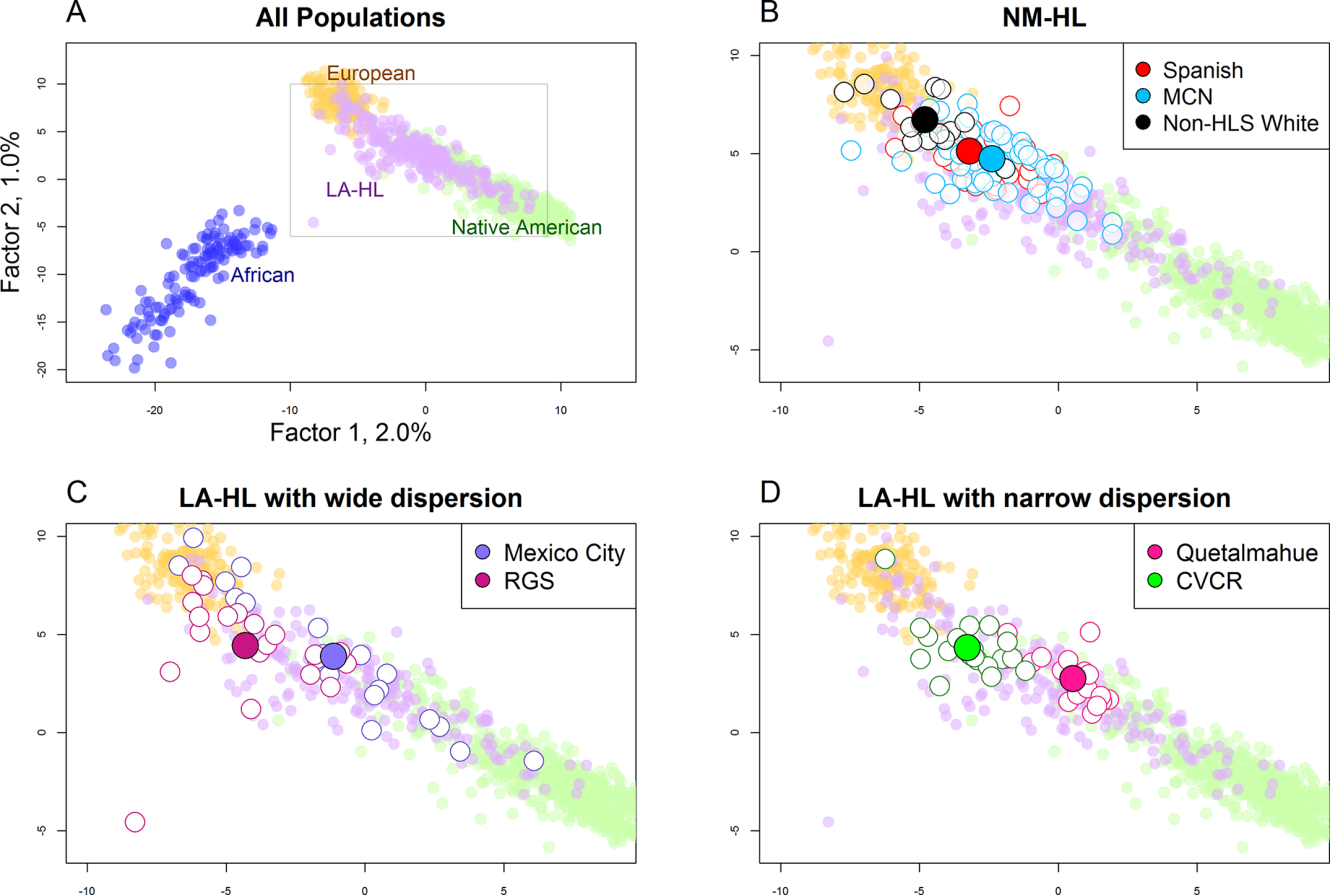
Scatter plot of PC1 and PC2 color-coded by geographic region. A. All individuals. B, C, and D show the HL portion of the plot. B. NM-HL subgroups. The outline color for the small circles show Spanish (red), MCN (blue), and one non-HSL and White parent (black). Large circles show mean PC1 and PC2 values for each subgroup. The pattern is similar for the All Spanish and All MCN subgroups (not shown on the plot). C. Mexico City and RGS. D. Quetalmahue and CVCR.

Fig 1A is a scatter plot of values for the first two PCs for our full sample of worldwide populations. Both factors separate individuals from Africa, Europe, and the Americas, and both are strongly correlated with European ancestry (R^2^_PC1_ = 0.88; R^2^_PC2_ = 0.87) and Native American ancestry (R^2^_PC1_ = 0.90; R^2^_PC2_ = 0.85) in the Native American and LA-HL samples. HL individuals are dispersed between the European and Native America clusters. Several HL individuals are also located relatively closely to the African cluster, most notably a single individual from the Brazilian RGS sample.

Fig 1B highlights the NM-HL individuals. Although there is overlap in PC values between individuals in different subgroups, individuals from the MCN subgroup (blue outline) are more widely dispersed, and they cluster closer to Native Americans than other subgroups. Four MCN individuals stand apart from the others in their relative proximity to the African cluster. Individuals with a non-HSL White parent cluster nearer to Europeans (two fall within the European cluster), as do, to a lesser extent, Spanish individuals. The mean PC1 values differ between the three subgroups at the 0.05 level. The patterns are similar for the all-MCN and all-Spanish subgroups (not shown).

Fig 1C and 1D highlight two LA-HL samples with high dispersion (Mexico City and Brazilian RGS) and two samples with low dispersion (Chilean Quetalmahue and Costa Rican CVCR) for both PC factors. In addition to the high degree of dispersion, individuals in Mexico City fall into distinctive clusters; six individuals cluster with Europeans, and, at the other extreme, a single individual clusters with Native Americans. This pattern of dispersion is potentially consistent with the existence of substructure within Mexico City. In contrast, individuals in the Quetalmahue and CVCR samples are relatively tightly clustered around their sample means.

### Hardy Weinberg equilibrium

**Fig 2:**
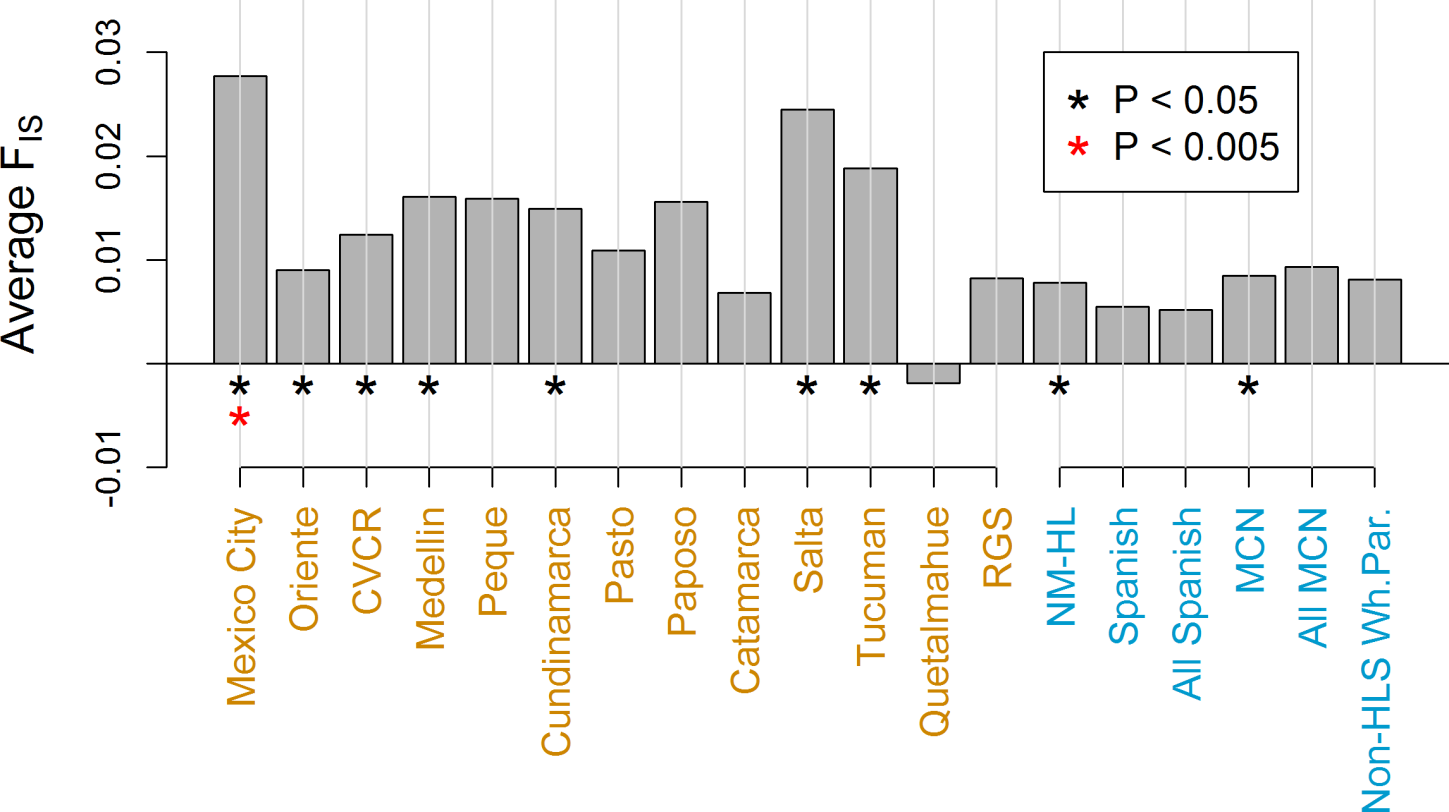
Average F_IS_. Positive values indicate a reduction of heterozygosity relative to that predicted under HWE.

Fig 2 shows the average F_IS_ values for the HL samples and the NM-HL ethnic subgroups. All samples except the Quetalmahue have positive F_IS_ values, suggesting that substructure may be common in HL populations throughout the Americas. In New Mexico, F_IS_ is significantly greater than zero at the 0.05 level in the NM-HL population as a whole and in the MCN subgroup. The latter result is consistent with the existence of additional substructure within the MCN subgroup. F_IS_ is comparatively low in the Spanish and All-Spanish subgroups.

### Genetic ancestry and one-time admixture

**Fig 3:**
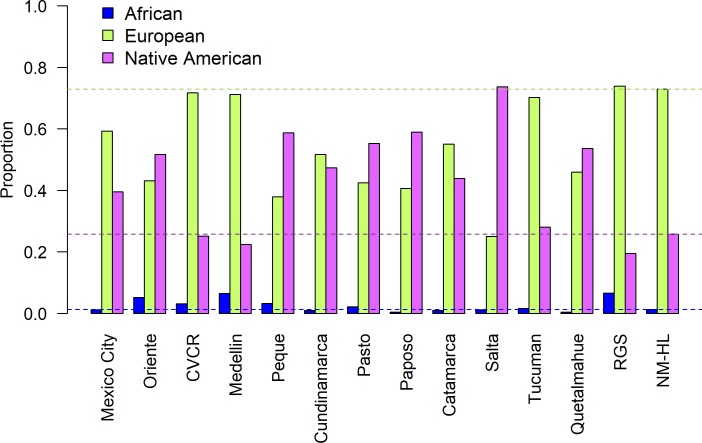
Bar plot of the mean individual-level ancestry for each sample averaged across 10 runs of structure at *K* = 10. Dashed lines show the mean Africa, European, and Native American ancestry for the NM-HL.

In the genetic ancestry analyses, the log probability of data, P(X|K), plateaued at *K* = 10. Two of the 10 clusters were specific to African and European individuals, and the remaining eight clusters captured substructure among Native American populations, most notable among relatively isolated populations in Brazil and Paraguay [23,31]. Fig 3 shows a bar plot of the mean individual-level African, European, and combined Native American ancestry for the HL samples. The blue bars show that African ancestry is broadly low, ranging from 0.4% in the Chilean Paposo to 6.6% in the RGS. The mean African ancestry in NM-HL is 1.3%.

All samples have comparatively high European and Native American ancestry. European ancestry ranges from a low of 25.0% in the Argentinian Salta to 73.9% in the Brazilian RGS. Conversely, Native American ancestry is lowest in the RGS (19.5%) and highest in the Salta (73.7%). Compared to the 13 LA-HL samples, the NM-HL sample ranks 2^nd^ highest in European ancestry (mean = 72.9%, range = 41.6–97.0%) and the 4^th^ lowest in Native American ancestry (mean = 25.8%, range = 2.5–57.0%). These ranges are similar to those reported from other studies of HL populations in the US Southwest [9,10].

**Fig 4:**
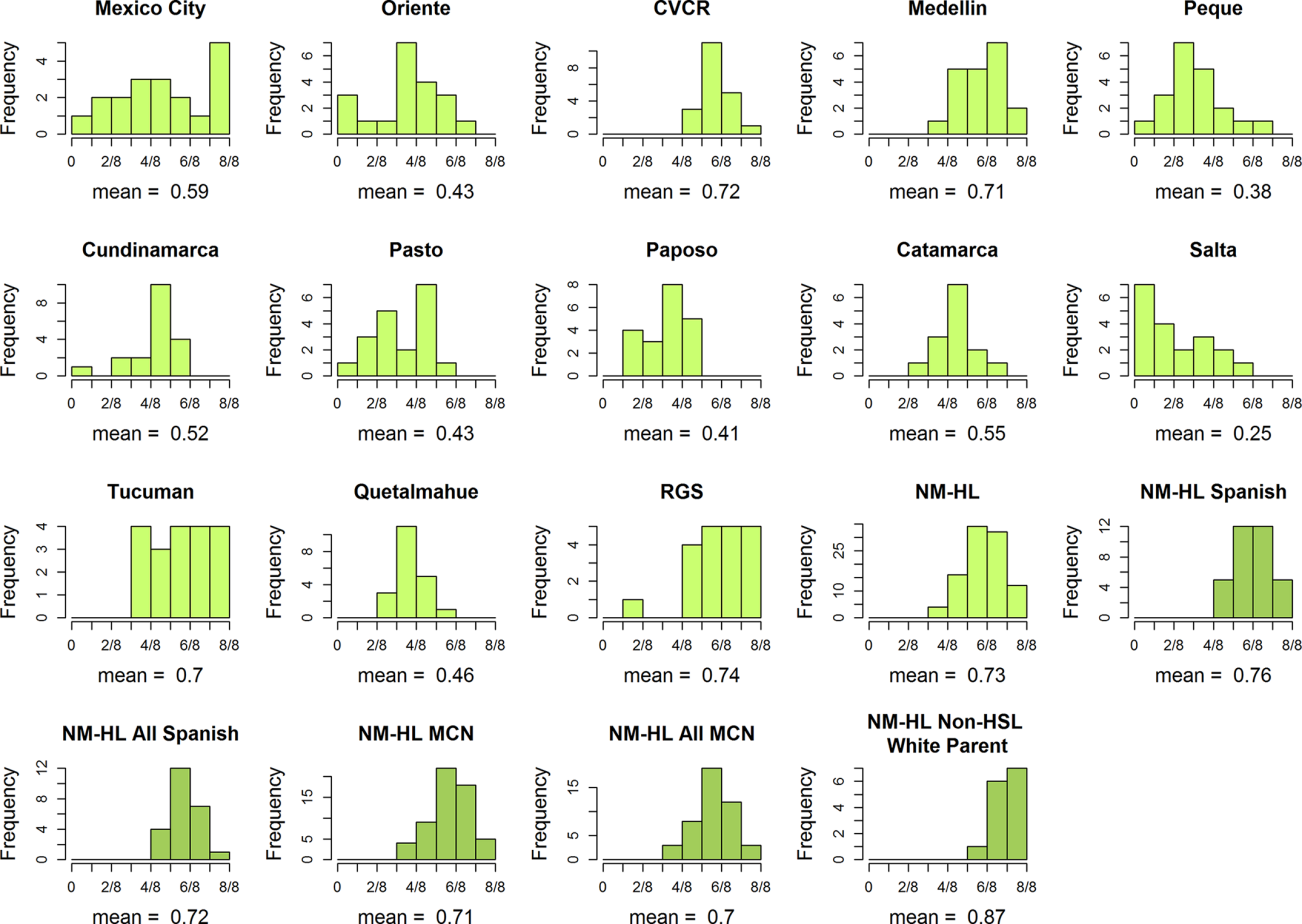
Histograms of individual-level European ancestry averaged across 10 runs of structure at *K* = 10. The mean European ancestry is listed below each plot. NM-HL subgroups are highlighted using dark green bars. Because African ancestry is low in the samples, the Native American ancestry distributions are effectively the complements of these European ancestry distributions.

The histograms of European ancestry in Fig 4 provide a more detailed picture of the degree of variation in ancestry within and among the samples (see S1 Fig and S2 Fig for the African and Native American ancestry distributions). In a handful of locations, the distributions are relatively narrow, e.g., the CVCR and Quetalmahue. In most locations, however, the distributions are broad and heterogeneous, most notably in the Mexico City (range = 2.6%-98%). This broad distribution in Mexico City is consistent with findings from a recent study of genomic diversity in other urban centers in Mexico [3].

For the NM-HL ethnic subgroups, the ancestry distributions are consistent with the PCA results in showing that the Spanish subgroup has a narrower range of European ancestry estimates (56%-94%) than the MCN subgroup (42%-94%) and a significantly higher mean European ancestry (76% vs. 71%, p < 0.05). The range of ancestry is narrower still for the subgroup with one non-HSL White parent (66%-97%), and the mean European ancestry (87%) is significantly greater than it is for the other two subgroups (p < 0.001). These results show that the genetic differences identified in the PCA analyses are the result of differences in continental ancestry among the NM-HL subgroups.

**Fig 5:**
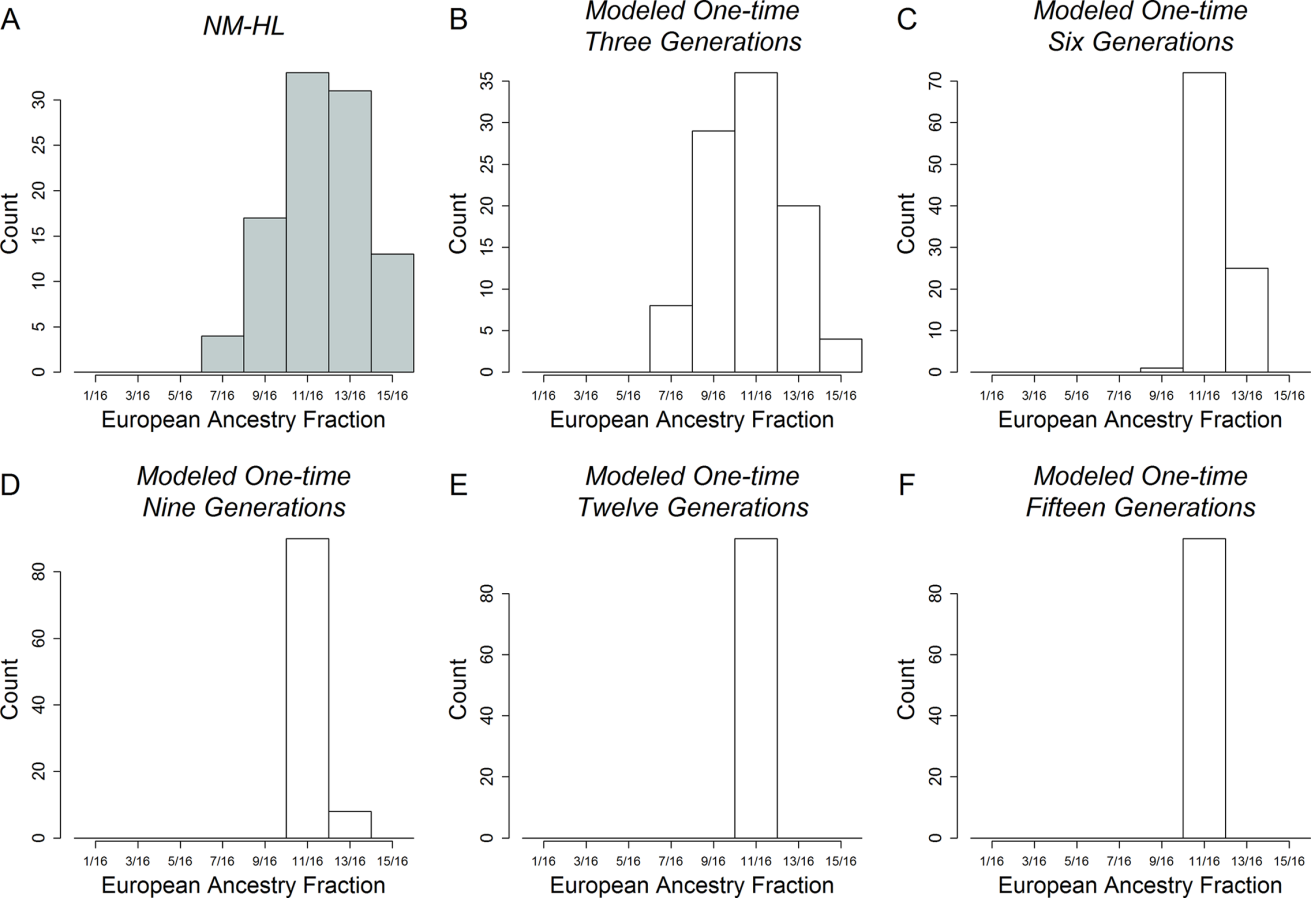
Histograms of NM-HL and model-based European ancestry. A. Empirical distribution. B-F. European ancestry distribution under a one-time model in which admixture occurred 3, 6, 9, 12, and 15 generations before present.

Fig 5 compares the European ancestry distribution for the NM-HL to distributions generated under one-time models of the admixture process with onset times at 3, 6, 9, 12, and 15 generations before the present. The plots show that the observed level of variation in ancestry in the NM-HL sample exceeds that produced under one-time admixture events occurring any time prior to three generations before the present. These results support findings from the PCA, HWE, and social-group identity analyses. We reject the hypothesis of one-time admixture in NM-HL. Furthermore, if admixture began between the 15^th^ - 19^th^ centuries in the 13 LA-HL samples, we reject a one-time model in them as well, with the possible exceptions of the CVCR and Quetalmahue.

### Credible region analyses

The sample sizes are relatively small for many of the HL groups (average = 25), and the 90% credible regions for individual estimates (from STRUCTURE) are fairly broad, averaging 6% (between low and high) across individuals for African ancestry, 23% for European ancestry, and 18% for Native American ancestry. Because these ranges are so broad, to confirm our conclusions about one-time admixture and to further explore the existence of substructure, we used the 90% credible regions to test the hypotheses that individuals in each HL sample had 0%, 100%, and the sample mean African, European, and Native American ancestry percentage. The results are shown in S2 Table. In the NM-HL sample, the 90% credible region excludes 0% African ancestry for only two individuals, with 14.6% and 12.2% African ancestry respectively, both of whom identify as MCN. The credible region also excludes 100% African ancestry for all 98 individuals. For European ancestry, the 90% credible region excludes 0% for all 98 individuals, and for all but six individuals, it excludes 100%. Of the six individuals, one is in the Spanish (and All Spanish) subgroup, and the remainder have a non-HSL White parent.

The 90% credible region for 17 NM-HL individuals fell above the sample mean (indicating relatively high European ancestry); of these individuals, three identified themselves and their parents as Spanish, four identified themselves and their parents as MCN, and nine had a non-HSL white parent. The credible region for 18 individuals fell below the sample mean (indicating relatively low European ancestry). Of these individuals, three identified themselves and their parents as Spanish, 11 identified themselves and their parents as MCN, and none had a non-HSL White parent. The results are similar (but complementary) for Native American ancestry. These results provide further support for the existence of substructure related to Spanish vs. MCN ethnic subgroup identity in NM-HL. They also confirm that admixture between non-HSL White and NM-HL individuals is common in the state, which is clearly inconsistent with one-time admixture.

Turning to the LA-HL samples, the 90% credible region excluded 100% African ancestry for all individuals in all HL samples, and it included 0% for all individuals in five of the samples. These results confirm that African ancestry is relatively low in these HL populations. European ancestry was relatively narrowly distributed in the CVCR and Quetalmahue. In these locations, the 90% credible region for ≥ 90% of individuals contains the mean European ancestry of the samples, and, except for one individual in the CVCR, the 90% credible regions exclude both 0% and 100% European ancestry for all individuals. The numbers are similar for Native American ancestry in these samples. The results are potentially consistent with one-time admixture in these two samples.

The credible region analyses confirm that European and Native American ancestry distributions are much more heterogeneous in the remaining HL samples. In Mexico City (n = 19), European ancestry spans 2.6–98%, and the 90% credible region excludes the sample mean for the majority of individuals (S2 Table). The credible region contains 0% European ancestry for 2 individuals and 100% European ancestry for four individuals. In the Argentinian Salta, the 90% credible region contains 0% European ancestry for six of the 19 individuals. In the Brazilian RGS, already highlighted for their high African ancestry, the 90% credible region contains 100% European ancestry for five of the 20 individuals. These results imply that substructure related to genetic ancestry is ubiquitous in admixed populations throughout the Americas.

## Discussion

Our results indicate that substructure related to genetic ancestry is ubiquitous in admixed populations throughout the Americas. Such substructure is potentially an important confound in studies that exploit admixed populations to identify the genetic component of disease, e.g., through genome-wide association and admixture linkage disequilibrium [32]. Substructure is also important in epidemiology and social science studies that seek to identify and eliminate racial and ethnic disparities in health and social welfare. It is important to recognize that group-level identity and its social and health-related correlates may vary widely among peoples that are commonly placed into a single category by the OMB, e.g., HL and African Americans.

### HL samples from Latin America

Though the studies of the LA-HL samples did not provide information about the racial or ethnic identity of the participants, we can use historical and demographic information to make inferences about the causes of substructure and the potential correspondence between social-group identity and genetic ancestry in these samples. We concentrate on two samples, one showing high dispersion in genetic ancestry (Brazilian RGS) and one showing low dispersion (Costa Rican CVCR).

In Brazil, the Instituto Brasileiro de Geografia e Estatıstica (IBGE) recognizes five categories of self-described social-group identity that are primarily based on phenotypic traits, including skin color: White, Brown, Black, Yellow, and Indigenous [33]. In 2008, the first three categories comprised 99.1% of the Brazilian population. In the Rio Grande do Sul, which comprises 5.7% of the population of Brazil, that number is 99.5% (White 80.8%, Brown 13.8%, Black 4.9%). Pena et al. [34] demonstrated that the three groups differed with respect to African, European, and Native American ancestry proportions estimated from 40 DNA polymorphisms. Mean European ancestry in individuals that identified as White, Brown, and Black, respectively, was 86%, 44% and 43%. Many individuals that identify as White had almost 100% European ancestry. Respective mean African ancestry values for the three groups were 5%, 44% and 46%. For the Brown and Black groups, African ancestry spanned 0–100%. This pattern of correspondence between social identity and genetic ancestry is consistent with studies showing that mating is assortative with respect to “color”-based social identity categories in Brazil [35].

In our analyses, European ancestry in the RGS ranges broadly from 14–97%, and the 90% credible region encompasses 100% for five individuals. Native American ancestry ranges from 3–40%, and the 90% credible region spans 0% for eight individuals. African ancestry was particularly heterogeneous; the 90% credible region spans 0% for 17 of the 20 individuals. In these individuals, the mean African ancestry is 1.8%. For the other three individuals, mean African ancestry is 33.6%, and it ranges from 17.2–58.7%. These results are consistent with those from Pena et al. [34], and they strongly suggest that the RGS sample is structured with respect to genetic ancestry.

In the sample from the Central Valley of Coast Rica (CVCR), the low dispersion in European and Native American ancestry, combined with the fact that the 90% credible region contains the sample-mean ancestry for all but two individuals (see Fig 2D, S2 Table), is consistent with one-time admixture occurring multiple generations in the past. This scenario is consistent with historical information documenting admixture between males from Spain and a small group of Native Americans females beginning in the 16^th^ century, followed by relative isolation [36]. This history is also consistent with findings from recent genetic studies of low levels of mtDNA diversity (comprised of 83% Native American haplogroups) compared to other Latin American populations [37].

### HL sample from New Mexico

We have more direct evidence for ethno-racial substructure in NM-HL. Most NM-HL individuals identify themselves and their parents as belonging to one of two groups; these groups broadly conform to a region-specific narrative that distinguishes relatively recent immigrants from Mexico from individuals whose families have resided in New Mexico for generations and who often emphasize their Spanish heritage. These groups differ from each other with respect to continental ancestry. Epidemiological and social science studies in the region should take this substructure into account, and they should work with individuals and scholars in local communities to identify the social groups that are relevant to New Mexicans. Such research is especially important given the potential lack of congruence between region-specific vs. national-level, e.g., OMB, racial and ethnic categories [38].

Fourteen percent of NM-HL individuals reported having a non-HSL White parent. Equal numbers of these individuals identified as Spanish and MCN (6 each). If such admixture has occurred for several generations, it may have eroded even larger ancestry differences that may have once existed between the subgroups. In fact, European ancestry in MCN is higher than it is in other Mexican and Mexican American populations, including those located near the US border [39–42].

These findings and interpretations must be tempered by the fact that the questions we used to elicit information about identity were not perfectly-suited to NM-HL. The first two terms in the Mexican-Chicano-New Mexico category conflate nationality and socio-political identity. Furthermore, the term “New Mexican” may have been associated by some participants with the region-specific term “Nuevomexicano,” which is typically associated with Spanish heritage [8]. Additionally, of individuals that identified as Spanish, 79% chose “Other specified Spanish origin (including European)” compared to 21% for “Spanish, Hispanic, or Latino, not otherwise specified.” The infrequent choice of the latter category may reflect that fact that it combines the potentially region-specific term “Spanish” with broader, more inclusive terms “Hispanic” and “Latino.” These identity-related questions highlight the broader problem of capturing how people conceive group-level identity in different regions of the world, especially when attempting to use generalized terminology that may be inappropriate or meaningless in a particular region.

In this vein, in 2009, the Institute of Medicine (now the National Academy of Medicine) recommended that health disparities researchers include lists of locally relevant fine-grained ethnic subcategories when collecting data [43]. To assist researchers, they compiled a list that separates the terms “Mexican” and “Chicano” into separate categories and includes region-specific terms such as “Nuevo Mexicano.” Regional studies, including ours, would benefit from the inclusion of such lists.

We also note that for the NM-HL sample, our European ancestry estimates are higher, and our Native American ancestry estimates lower, than those from other studies of the region [9,10]. The variation in estimates might reflect differences in sampling methods, e.g., exclusion of individuals with non-HL White parents. It might also reflect differences in the types of markers used in the studies (autosomal microsatellites vs. SNPs). Autosomal microsatellite loci are prone to allelic dropout, which could lead to errors in allele frequency estimation and tests of HWE (e.g., inflating F_IS_). We eliminated loci and individuals with high amounts of missing data, but it is possible that dropout affected some of our F_IS_ results.

## Conclusions

We found that racial and ethnic identify among NM-HL conforms to a region-specific narrative that distinguishes recent immigrants from Mexico from individuals whose families have resided in New Mexico for generations and who emphasize their Spanish heritage. Our analyses of NM-HL and 13 Central and South America HL samples suggest that genetic substructure is ubiquitous in admixed populations in the Americas, and that this substructure may be related to variation in social identity. This substructure may confound attempts to identify disease-causing genes and to understand the social causes of variation in health outcomes and social inequality.

## Supporting information

S1 Fig African ancestry. (TIF)

S2 Fig Native American ancestry. (TIF)

S1 Table Ethnic subgroup participant and parents. (XLSX)

S2 Table Credible region analyses. (XLSX)
