# Supplementary figures and images for: Social-group identity and population substructure in admixed populations in New Mexico and Latin America
Source: PLoS One. 2017 Oct 4;12(10):e0185503. doi: 10.1371/journal.pone.0185503 (PMC5627912; doi:10.1371/journal.pone.0185503)

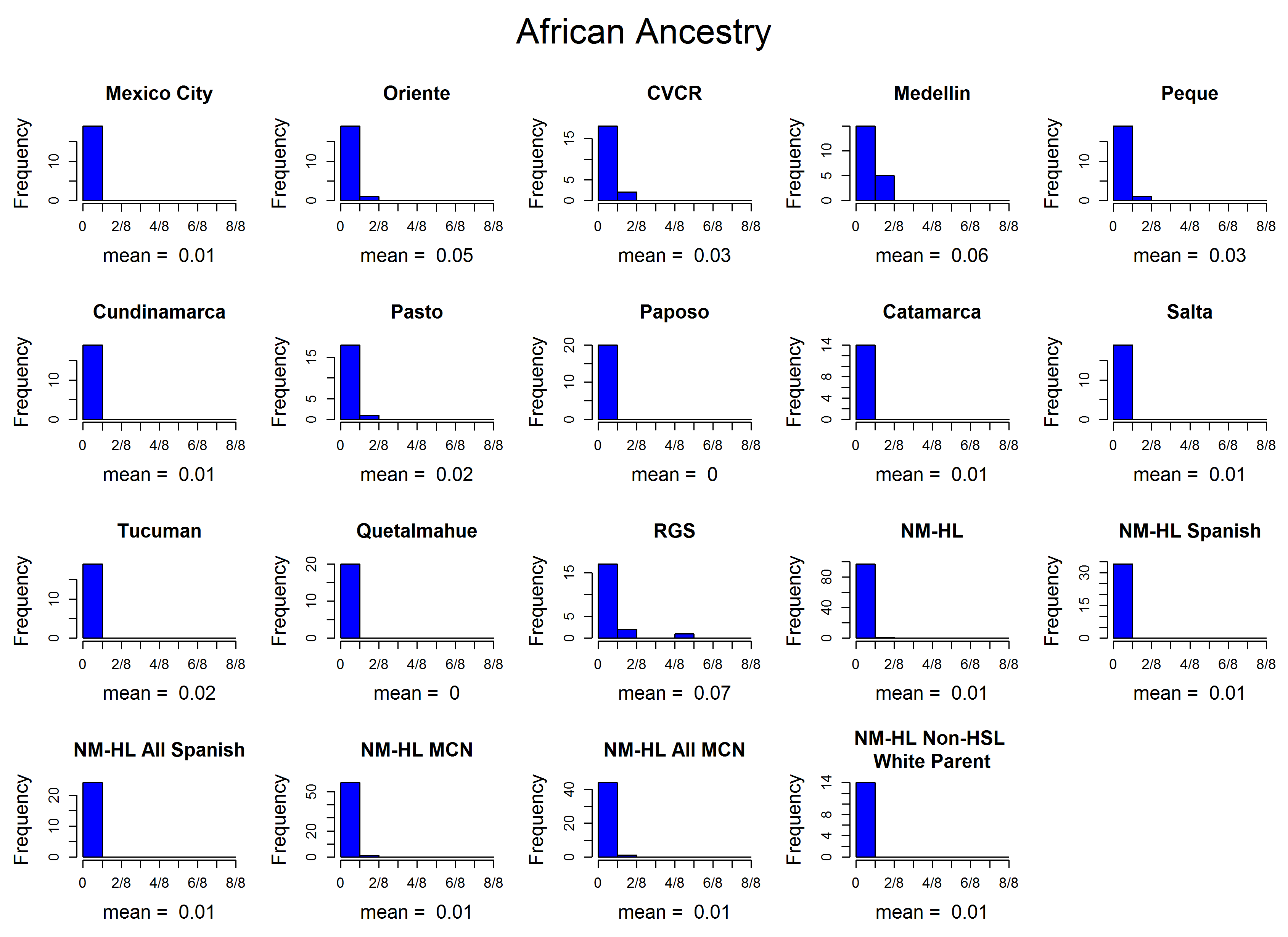

Supplement: S1 Fig — (TIF) [file pone.0185503.s001.tif]

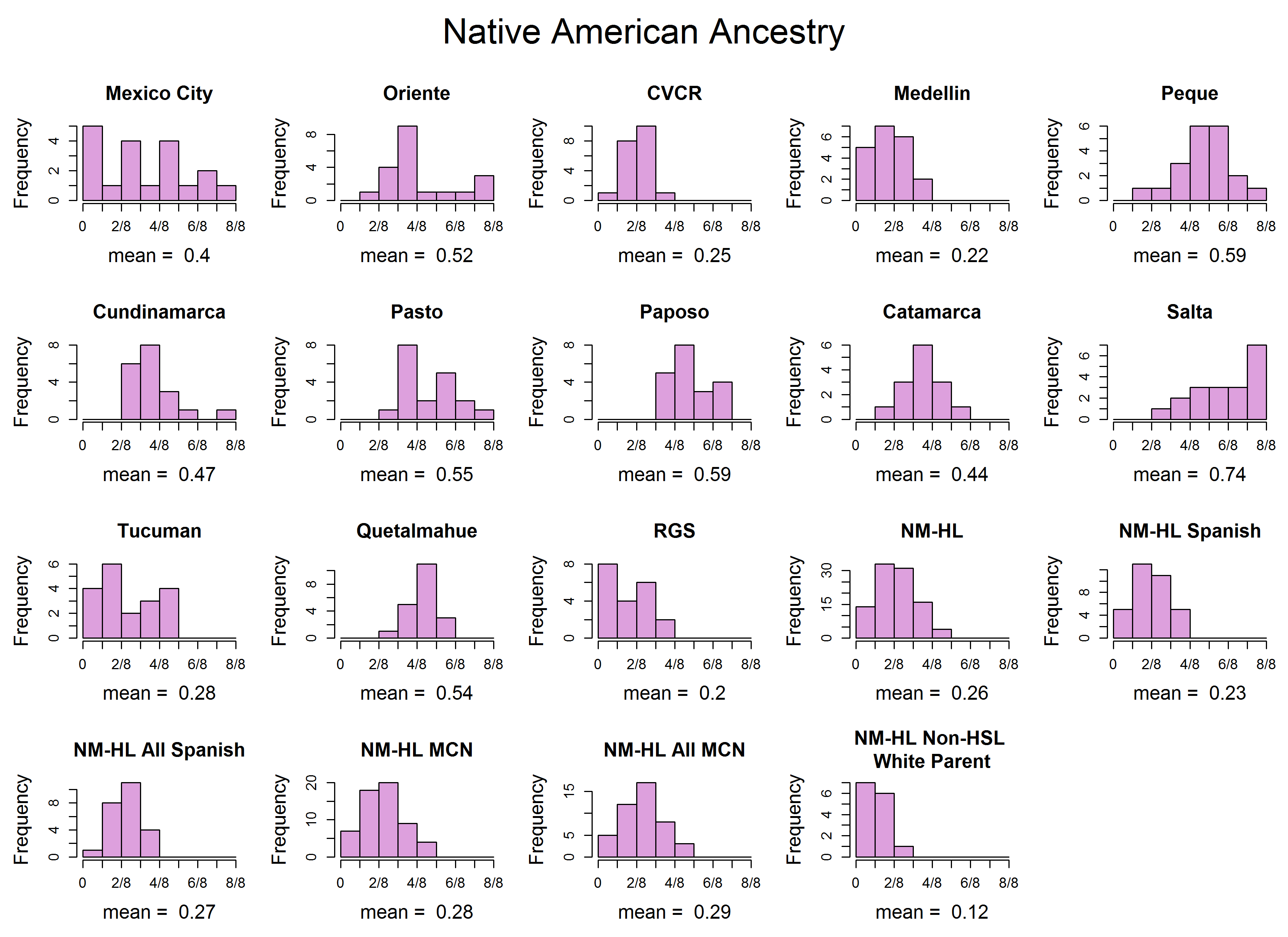

Supplement: S2 Fig — (TIF) [file pone.0185503.s002.tif]
